# Fruitfulness of Foreigners in Boston

**Published:** 1846-09

**Authors:** 


*Fruitfulness of Foreigners in Boston.*—In Broad street, a low, water-edge street in Boston, in which Irish families are inconveniently thick, Mr. Shattuck, author of the Boston Census, says there are 2131 inhabitants—and that the births amount to *one in fifteen* of the whole population. This prodigious fecundity, he continues, is a most remarkable characteristic of our foreign population, and generally prevails throughout the city. It is, no doubt, one of the principal causes of the increase in the number of deaths among children under five years of age.—*Boston Med. and Surg. Jour.*

